# Main risk factors for diabetic foot

**DOI:** 10.1186/1758-5996-7-S1-A26

**Published:** 2015-11-11

**Authors:** Marília Klein Reis, Júlia Scaravelli Mario, Mari Cassol Ferreira

**Affiliations:** 1 Universidade Comunitária da Região de Chapecó, Chapecó, Brazil

## Background

Diabetic foot is a chronic complication of diabetes mellitus (DM) that may manifest as neuropathy, vasculopathy, osteoarticular involvement and infection. Foot injuries are among the most serious and costly complications of diabetes. Foot care programs can reduce the occurrence of injuries by 50%.

## Objectives

Assess the determinant risk factors of diabetic foot in DM patients assisted in the public health service of Chapecó-SC.

## Materials and methods

Descriptive, cross-sectional study evaluated 130 diabetics type 1 and 2, over 18 yrs. old. The survey instrument adapted from “Rastreamento e avaliação precoce dos fatores de risco e prevenção do pé diabético”, developed by SBD/DF and Grupo de Pé Diabético do Brasil, was applied to analyze neuropathic symptoms, feet clinical inspection, loss of protective sensation (LPS), peripheral arterial disease (PAD), ulcers, amputation and risk classification – 0: without LPS and PAD, 1: presence of LPS±deformities, 2: PAD±LPS, 3: previous ulcers and/or amputation.

## Results

Samples evaluated showed 99.2% of DM patients had DM2, 70% female, age 64.68±11.45 yrs. old, disease duration 10.4±8.5 yrs. and 17.7% of insulin users. Mean HbA1c was 8.02±2.19%. Neuropathic symptoms were reported by 75.3% of patients, predominantly burning, numbness or tingling. The most prevalent clinical findings were dry skin, cracks or fissures (76.2%), ungueal mycosis (33.8%) and calluses (31.5%). Inappropriate footwear use was seen in 74.6% of subjects. LPS was found in 28.5%, and 73.3% of these had HbA1c>7% (p=0.069). LPS was detected in 80% of patients who had previous ulcers (p=0.023). The majority (75%) of patients with involvement of thin fibers (burning or pain and/or thermal sensitivity decreased) had diabetes for less than 10 yrs., which is shown as a premature injury. While in 52,1% of patients with damage in thin+thick fibers (thin fiber+irregular monofilament exam and/or decreased vibratory sensation) had more than 10 yrs. of disease (p=0.031). PAD signals were observed in 20% of the sample. Regarding the risk classification, 57.7% were classified as risk 0, 20.8% risk 1, 16.9% risk 2 and 4.6% risk 3.

## Conclusion

Data confirms the impact of timing in diabetes evolution and uncontrolled glycaemia, also shows the onset of thin fibers signs of neuropathy occurs early before the LPS, serving as a warning for diagnosis and treatment in this evolution phase of peripheral neuropathy, which is the main risk factor for diabetic foot.

